# The Shame of Addiction

**DOI:** 10.3389/fpsyt.2013.00120

**Published:** 2013-10-08

**Authors:** Owen Flanagan

**Affiliations:** ^1^Department of Philosophy, Duke University, Durham, NC, USA

**Keywords:** addiction, alcoholism, substance-related disorders, shame, blame, guilt, willing addicts, resigned addicts

## Abstract

Addiction is a person-level phenomenon that involves twin normative failures. A failure of normal rational effective agency or self-control with respect to the substance; and shame at both this failure, and the failure to live up to the standards for a good life that the addict himself acknowledges and aspires to. Feeling shame for addiction is not a mistake. It is part of the shape of addiction, part of the normal phenomenology of addiction, and often a source of motivation for the addict to heal. Like other recent attempts in the addiction literature to return normative concepts such as “choice” and “responsibility” to their rightful place in understanding and treating addiction, the twin normative failure model is fully compatible with investigation of genetic and neuroscientific causes of addiction. Furthermore, the model does not re-moralize addiction. There can be shame without blame.

“Dear friends, be men; let shame be in your hearts … Among men who feel shame, more are saved than die.” Ajax to his troops in Homer’s *Illiad*

## The Twin Normative Failure Model of Addiction

I propose a twin normative failure model of addiction in which the self-regarding reactive attitudes of bewilderment, disappointment, and shame play a constitutive role^1^. The addict cannot pass her own survey because she self-interprets, and self-interprets correctly, that she fails to execute normal powers of effective rational agency, she decides not to use and uses; she also fails to live up to the hopes, expectations, standards, and ideals she has for a good life for herself because of her addiction. It is possible and desirable to understand addiction as a normative failing in both these respects, one of rational effective agency, the second of moral quality broadly construed, without also moralizing addiction. Recognition of these twin normative failures is a powerful source of desperation and motivation to heal on the part of the addict and an immensely valuable tool for the therapeutic community to keep in view and use non-moralistically, as it tries help the addict to heal. In earlier times, it may have been that most addicts died for reasons related to addiction. Now, perhaps, with much deeper knowledge of the causes, components, effects, and nature of addiction we can make Ajax’s maxim true for addicts: “Among men who feel shame, more are saved than die.”

I intend the twin moral failure analysis to be true to the perspective of the addict, his close relations, as well as from the perspective of the professional and non-professional therapeutic communities that work and live with addicts. I also intend the analysis to describe normal and reliable features of addiction. The exceptions, the cases where there is no shame on the part of the addict descriptively, and where shame would be unwarranted normatively involve abnormal psychiatric conditions, e.g., schizoid personality disorder, or unusual social conditions where there are no choice-worthy options for a good human life, or where the addict has a certain social status, social permission (opiate addicts in hospice), and/or financial resources to be an addict with impunity.

To many, the view will seem too strong in two respects. First, some addicts are willing and not ashamed, so the view is descriptively false. Second, the view is reactionary: despite my claim that the twin normative failure model does not moralize addiction because it acknowledges shame without endorsing blame, it nonetheless returns us to the view that addiction is a moral failing. After spelling out the view more fully, I reply to these and several other objections.

## Addiction is a Normative Disorder

In a seminal paper in philosophy and cognitive science, “Intentional Systems,” Dennett (1) distinguished between three stances we commonly take toward ourselves and other human beings, the “intentional stance,” the “design stance,” and the “physical stance.” From the perspective of the intentional stance we deploy psychological or mental vocabulary to describe, explain, and predict our own mental states and actions and those of others. We think of ourselves and our fellows as “intentional systems,” as human individuals chock full of beliefs, desires, emotions, and goals. And we think of a particular integrated suite of embodied intentional states and dispositions as what makes an individual tick, what makes them who they are, what’s behind the personality and character they display. The design stance goes below the intentional or person level and uses concepts related to normal or proper function: drinking a cup of coffee involves a perceptual system that registers that there is a cup of coffee, a system that computes desire and eventuates in a decision to drink it, which sends a signal to the motor system to move the hand in the right way, lift-to-lips, and drink. The physical stance goes lower still to the level of actual physical realization. The physical stance is especially useful when there is breakdown of proper function: Ann drinks coffee through a straw because in the bike accident she badly sprained both wrists.

Higher levels depend upon and are implemented by the lower levels. One could take the physical stance description lower: sprains are implemented on wrists, specifically on ligaments, which are made of flesh, which is eventually, like everything else, implemented on bosons and fermions^2^. But higher levels also have emergent properties in one perfectly natural sense. Wiffle balls are round, but the molecules that compose them are not. A hard hit wiffle ball can cause a bruise, but a hard hit polymer atom in a wiffle ball cannot cause a bruise. Wholes have properties that their parts don’t. So too persons have properties that their parts, brain parts, and gene parts do not. Addiction is a person-level phenomenon. Neither brains nor genes are the sole cause of addiction; nor do brains or genes become addicted. That said genetic and neural malfunctions are clearly an important part of the explanation of how and why some people, some intentional systems, become addicts and suffer addiction.

At the intentional stance level, but not at lower levels, persons, whole persons, are normative creatures in two senses, one constituted by our power to assess or evaluate ourselves as rational or reason-responsive agents in a broad sense and to do what we decide is best to do all things considered; the other constituted by our power to assess or evaluate ourselves morally or from a moral point of view. Normative governance consists of two complex and highly interactive capacities, rational governance and moral governance. Rational governance refers to the capacity of persons to determine what we have reason to do, what makes sense to do given our aims, interests, and the way(s) the world is. Moral governance is the capacity to judge some of the things we do (or intend to do) as good or bad, right or wrong.

Our power to assess or evaluate ourselves as rational or reason-responsive agents is both episodic and diachronic. Episodically, at any one time or period in a life, there is always a set of possibly true answers to two sorts of questions. One set concerns what a person is doing here and now (at this time or within this period of time); the other set concerns why the person is doing it. As to the question of what I myself am doing, suppose what I am doing here and now is driving my car through the Holland Tunnel from Manhattan to New Jersey. As to why, the reason is that I believe that for someone in my physical position, in lower Manhattan, with a car, and wanting to get to Jersey, the best or most convenient way to get to Jersey is to take the Holland Tunnel. My physical position and my desire to get to Jersey give me good reason to drive through the Tunnel. I am responding to that reason in driving through the Tunnel. Normally, when a person has reason to Φ, she Φ’s. Addicts are puzzling including to themselves. When it comes to their drug of choice (DOC), what David Foster Wallace types as “the Substance,” they are performatively inconsistent (2). They resolve not to use and use. Many addicts report that the resolve and the action that undermines it occur at the same time, virtually in the same instant.

Our lives are also lived and led in diachronic psychological space and not just in the moment or in brief episodes. We experience ourselves as someone who was there in the past and we conceive ourselves as someone who will be there in the further future, short-term and long-term. We experience ourselves as not just being alive, but as having a life to lead, self-direct, or control, where a life is conceived as “the sum of one’s aspirations, decisions, activities, projects, and human relationships” [(3), p. 5]. Most of us probably do not have a blueprint for our whole life, what Rawls (4) called a “life plan,” but we do have for ourselves multifarious projects and plans nested together in various, possibly ever-adjusting, relations of priority and expansiveness. For many, most, perhaps all of us persons, we develop a narrative self-interpretation of ourselves as persons and perpetually evaluate how well we are doing in becoming who we aim to be and in accomplishing what we aim to accomplish. A basic way in which to understand the inter-relationships between our past, present, and future is to conceive of the lives we lead “as an unfolding story” (5, 6)^3^. There comes a time in every addict’s life when he comes to see that his “self-represented identity” and his “actual full identity” are on divergent paths, likely far apart, possibly inconsistent (7)^4^.

Finally, our lives are lived in social space as gregarious social animals. Most humans have natural desires for companionship and most of us recognize, even if only inchoately, that we cannot survive, develop ourselves as persons, or live good lives, that is, lives which are happy as well as meaningful and fulfilling, without situating ourselves in complex socio-moral relationships with other persons. And despite wide cultural variation in the exact norms governing social practices we all typically engage in normatively governed practices of “lending and borrowing, promising and consenting, buying and selling, making friends, entering into marriage, establishing a family, offering and accepting aid, and so forth” [(8), 20]. Reliably gaining the goods associated with these practices – security, self-esteem, self-respect, social trust, friendship – involve broadly moral evaluation diachronically by oneself and others^5^.

This picture of levels of explanation and of persons as normative beings in the twin – rational self-interpretation and self-control sense, what I call, the “rational effective agency sense”, and “the moral sense” – has implications for thinking realistically and humanely about addiction. Addiction is a person-level disorder – actually a person-in-a-particular-social-world disorder – in which there is failure of normative governance by rational norms of narrative or biographical integration and moral norms. The result is first bewilderment on the part of the agent that she has lost some of her normal capacities to direct her behavior by what she judges as her considered desires and reasons (for example, to drink like a normal person; not to drink to blackout), and eventually deep shame, as well as a host of other reactive attitudes, at the fact that she is not doing well by her own and often widely shared standards^6^. The model of addiction, the “twin normative failure model,” and of the addict, I propose rejects reductive models of addiction that claim that addiction is a design or physical level disorder. Addiction occurs in creatures with brains and genes (and bosons and fermions) but it is not a disorder of brains and genes (or bosons and fermions). It might involve disorders of either or both (9–11)^7^. Persons are addicts. Addiction is a person-level disorder, a diachronic intra-personal and inter-personal disorder; it is a disorder of persons that involves normally and reliably shame in one’s own eyes, and thus losses of self-respect and self-esteem, as well as social shame, a sense that even if one is not actually seen for who and how one is by others, one would be judged weak, weird, undisciplined, untrustworthy, and scary if one were seen for how one is as a person-in-the-grip of an addictive behavior pattern. Addiction fully engages the reactive attitudes of the addict, even if neither he, nor his community, judges him harshly or moralistically^8^.

One consequence of my view of addiction and its relationship to the experience of being an addict is that it accepts rather than resists the idea that addiction really is, in the eyes of the addict, and those with whom she is in community, a normative problem. The addict has trouble with respect to her addiction putting her reasons, her best thinking, in reliable control of her actions, and she has trouble (perhaps for this reason) abiding by the moral norms upon which her sense of her own integrity and self-worth turns. It is disrespectful to the phenomenon of addiction, to addicts who experience their addiction as involving these twin normative failings, and to the wider community, which judges addiction as bewildering, sad, and shameful to deny that it is a straightforward normative disorder. It is equally extremely shortsighted and inhumane to think that the problem that an addict has is a straightforward problem that can be solved by a psychopharmacological intervention to stop the desire to use or the effects of using. What most don’t see because of the meager dialectical offerings – addiction is either a moral or a brain/gene disorder – is the prospect that one can see addiction as involving biographically interpretative assessment of one’s own reason responsiveness failings as well as moral failings without either the addict herself or her community moralizing and blaming her. The theory is that at the level of persons, social persons, addiction is a failure in two highly interactive normative systems at once. One can think this, just as one can think that addiction involves some choice and some responsibility without blaming the addict and moralizing addiction. Let me explain.

## DSM 5 on Substance Abuse and Addictive Disorders

Technically DSM 5, (12) like its predecessors, is only a diagnostic manual. It offers a classificatory scheme. DSM 5 taxonomizes and conceptually disciplines disorder. It doesn’t claim to explain (indeed it positively refuses to provide etiologies), or to offer treatment or therapeutic regimens. What DSM 5 is not agnostic about is that the symptom cluster it uses to diagnose substance-related disorders will yield to neuro-specification^9^. The manual is full of strong hints that it expects its symptom clusters to be filled in by neuro-specifics. We learn that all drugs of abuse have in common “direct activation of the brain reward system;” that people with “impairments of brain inhibitory mechanisms may be particularly predisposed to develop substance use disorders.” The section on substance abuse disorders, like the rest of DSM 5, is filled with “maybes” about connections to genes and the brain. The facts are that there must be such connections; humans are genetically endowed animals and our nervous systems are involved in everything we do. But it is a mistake to think that all properties of persons reduce to properties of parts, even especially important parts. I could argue that all the winks and nods to neuroscience (and to a lesser extent, to genetics) is part of the mindless cultural spread of neuro-enthusiasm (and what Rob Wilson calls “smallism”), which although true, would distract from my aim to describe what addiction is in a way that is true to the phenomenon and that is also therapeutically useful, recognizing the many roles that shame and related reactive attitudes can and do play in healing from addiction.

It is also a mistake – a related one – to think that all the essential features of addiction are features that can be revealed in non-human animal models of addiction. The brain reward system of non-human animals has interesting similarities to the human reward system, but the social ecologies of mice and humans are entirely different, as are the capacities served by culture and an enormous prefrontal cortex. A rodent cannot consciously resolve, possibly in consultation with fellow mice, to refrain from consuming a drug because its life is not going well, because it is causing communal harm. A rodent cannot relapse, and then regret and feel ashamed or guilty for its failure to maintain abstinence. Animal models may teach us about how dangerous and imprudent it can be to suddenly reverse preferences over time, but the full character of human addiction is no mere preference reversal or oscillation. It normally involves an interpretation and evaluation of oneself as having let oneself down; of having broken promises to one’s own self (and others). Non-human animals, at least the ones studied in addiction labs, are not self-interpretatively normed. They don’t see themselves as leading a life. Nor are they moved by thoughts of what counts as a good person/rodent, nor puzzled or disturbed by feelings of guilt, shame, and embarrassment. The moral virtue or value of self-control and of responsibility for self is irrelevant to animal addiction. But with a human being, a person’s social relationships, the effects of his actions on others, his loyalties and friendships, his trustworthiness, are deeply relevant to his being an addicted human being^10^.

What is really interesting is that the DSM 5 criteria for substance-related and addictive disorders are very plausible and never mention, not once, the brain or genes. What they do mention, and rightly so, are normative impairments. Here is the list of diagnostic criteria for alcohol-related disorders [(12), pp. 490–491], which are representative of the substance abuses overall:

1. Alcohol is often taken in larger amounts or over a longer period than was intended.
2. There is a persistent desire or unsuccessful efforts to cut down or control alcohol use.
3. A great deal of time is spent in activities necessary to obtain alcohol, use alcohol, or recover from its effects.
4. Craving, or a strong desire or urge to use alcohol.
5. Recurrent alcohol use resulting in a failure to fulfill major role obligations at work, school, or home.
6. Continued alcohol use despite having persistent or recurrent social or inter-personal problems caused or exacerbated by the effects of alcohol.
7. Important social, occupational, or recreational activities are given up or reduced because of alcohol use.
8. Recurrent alcohol use in situations in which it is physically hazardous.
9. Alcohol use is continued despite knowledge of having a persistent or recurrent physical or psychological problem that is likely to have been caused or exacerbated by alcohol.
10. Tolerance, as defined by either of the following:
  - A need for markedly increased amounts of alcohol to achieve intoxication or desired effect.
  - A markedly diminished effect with continued use of the same amount of alcohol.
11. Withdrawal, as manifested by either of the following:
  - The characteristic withdrawal symptoms for alcohol (refer to Criteria A and B of the criteria set for alcohol withdrawal, pp. 499–500).
  - Alcohol (or a closely related substance, such as a benzodiazepine) is taken to relieve or avoid withdrawal symptoms.

## The Phenomenology of Addiction

DSM is wisely explicit, despite the thick vein of neuro-enthusiasm that courses through it, that substance abuse involves “*impaired control*” and “*social impairment*.” The language it uses to depict addiction is the language of the intentional stance. The addict has trouble carrying out his “intentions” (1); he “desires” to control using and makes “persistent efforts” to do so (2); he spends “a great deal of time in activities necessary to obtain alcohol” (3); he has “persistent and recurrent social and inter-personal problems” (4–7). Understood in this way, it looks as if DSM 5 embeds something in the vicinity of the twin normative failure model in its description of the typical cluster of features that define, characterize, or constitute addiction.

But, truth be told, DSM 5 is unclear about whether it considers these features merely symptomatic (often that seems the implication) the way fever is with a flu, or whether it understands these features as constitutive, as necessary, or as part of the typical psychological profile of addicts. For any mental disorder we can distinguish among its causes, its components or constituents, and its consequences. Because humans are self-interpreting animals, classifications, components, and consequences are absorbed, recorded, reflectively assessed, and they change the person being classified, who as a consequence of his disorder and his comprehension of it causes myriad further effects on himself and others. If one examines the vast literature comprised of memoirs of addiction (13) as well as writings by therapists who work with addicts, most every entry on the DSM 5 list will be familiar as constitutive of addiction. When the addict feels shame before his own eyes, when he observes his control failures, when he understands that he is an addict not living up to his own standards or best interests, he changes, for better or worse. The story of who he is, what he is like, how well he is doing in the task of accomplishing what he intends, of living well, absorbs ever new material. There is narrative self-interpretative adjustment. Since narrative is partly constitutive of the self, he changes (7, 14). For the addict, along the descent depicted by DSM 5 steps 1–11, he is bewildering to himself, possibly terrifying – he can’t get a grip – he’s a disaster, a train wreck harming himself and those he loves. This first personal normative assessment does really capture the shape, the texture, and the phenomenology of addiction. Is there a mistake? No. Indeed, it is only by understanding these normative failings that the addict shows himself the self-respect he deserves as a person and leverages, normally with communal assistance, his remaining powers of agency to get back on track and repair himself as well as the situations and relations that have been damaged along the way.

Despite the fact that the DSM 5 list of symptoms includes failures in self-control and in reliability – in behavioral failures with respect to norms – it is missing any reference to normative feeling states, to reactive attitudes associated with these behavioral failures. The addict has “cravings,” and greater “tolerance” involves changes in the “effects” of the DOC. He continues to use despite “knowledge” that using causes him problems, etc. But there is nothing in 1–11 about the way the addict experiences his failures to rationally effectively and morally guide his life through and past the allure of alcohol, which consistently undermines, possibly defeats his attempts to live well and to be well^11^. Interpreting the DSM 5 criteria to include these reactive attitudes or extending its criteria to include the feelings associated with the twin normative failures it almost always involves, would capture better the psychology of addiction than any purely behavioral, neuroscientific, or genetic model.

## The Interpretative, Reasons-Responsiveness, Effective Agency Failure

The twins that fail in addiction are fraternal, not identical. What I call the interpretive, reasons-responsiveness, effective agency failure is really a set of failures that include cognitive mistakes such as minimization (I don’t drink too much; I just need to stop at *n*, where *n* > what a normal person would think is wise), and rationalization (you’d drink a lot too if you had my _____). One failure that is most familiar to both addicts and those in relation to them is a failure to execute rational control, to be able to execute rational plans, the failure to be in charge. The simplest way to put this point is in terms of the performative inconsistency mentioned earlier, which every addict understands as constitutive of his situation: I will/decide/pledge/promise to myself (possibly to others as well) that I will moderate or stop using; and then I use. P and not P. The failure is one of effective agency, of leading one’s life and not just tagging along for the addicted ride. The addict like everyone else sees himself as a being with hopes, projects and plans, responsibilities and obligations, friendships, and loves, as an historical, enduring being, possessed of long-range interests. But his own defective agency gums up the works, the work of being and becoming the person he aims to be. He fails at reliably enacting in-charge selfhood. If his DOC, the Substance, is available he loses normal self-control against getting lost in a preference oscillating and the preference-reversing moment or episode. He is bewildered and ashamed.

According to DSM 5, this sort of complex failure begins in mild addiction at step 1, where the substance abuser uses more that he at first intends. And it gets worse as the problem becomes more severe. It is sometimes hard to tell from behavior where exactly a drinker is in terms of loss of the ability to stop in a normal reasons-responsive way, i.e., by making an all things/future me considered judgment that they should moderate or stop, and then doing so. The *Big Book of AA* written in 1939, not a scientific work, recognizes correctly a certain kind of “heavy drinker,” who if he has reason to stop (the liver, the job, the spouse) stops. And much recent work confirms that many people who drink heavily, possibly in binges, possibly regularly, at some point, in their lives stop (15, 16, 43). But there is a type of drinker who seems not to be like this. They try to stop but can’t. Caroline Knapp’s, *Drinking: A Love Story*, Pete Hamill’s, *A Drinking Life*, and Charles Jackson’s, *The Lost Weekend* are powerful depiction of such lives.

The brain basis of addiction, according to animal studies, lies, in significant part, in the mesolimbic dopamine and brain reward system. It is possible that this area is compromised in humans, not only in opiate addiction, but in alcoholism as well. Suppose it is, and that therefore a compromised mesolimbic reward system is a necessary condition of human addiction. It does not follow that it is sufficient or that it is the only necessary condition (42, 44). In humans, addiction is constituted not only by craving, compulsive use, and “jonesing” for the Substance, which may be subserved primarily by the compromised mesolimbic reward system, it is also experienced and treated just in case the person experiences herself as unable to stop given that she has reason to (the personal and social costs mentioned in DSM 5); and that she is ashamed that she is not able to live as she judges to be good. This sort of self-regarding reactive attitude is I claim is a normal part of the phenomenology of addiction but not mentioned in DSM 5. It is implausible that human beings control against consumption impulsivity and imprudent preference reversal only by virtue of some sort of inhibitory mechanism in the brain reward system. If they did, then it might be plausible to say that addiction represents a disorder of that mechanism, and of nothing else. But human sources of inhibition and self-control are known to be many and various. Human powers of deliberation, self-assessment, and reason responsiveness are subserved by neural systems, especially in prefrontal cortex, that differ in organizational complexity from those of rodents. Furthermore, human linguistic capacities put us in unusual touch with communal norms and with communal reasons for abiding by those norms. To be sure, the addict has trouble making her reasons effective and this may have to do with damage to the circuitry in the mesolimbic areas or in the areas that connect prefrontal cortex to lower regions. But the facts are that addict has her reasons to stop, wishes to stop, but can’t. And it is this experience of the failure to execute effective agency, according to my argument, that is also constitutive of human addiction, although almost certainly not of rodent addiction^12^. A human addict cannot in a situation in which he is considering if he can and should refrain from drug consumption, regard himself “as waiting to discover or to observe in which direction he will be moved” [(17), p. 51]. To be counted by him as a decision of whether or not to refrain, the state of deciding must be thought to conform to some standard of possessing a good reason. The question “Will I abstain?” is unavoidably indistinguishable from “should I abstain here and now?” The total situation I am in as an addict confronts me, and sets the problem. I may try to take account of things about myself that I believe are not in my power to change (because I lack the means or skill to change) and those things I believe I can change. The anxiety, felt need, impulse, the craving, the sudden passion to consume, when they occur, may feel like something that descends upon me. I may try to double back upon myself and think of myself as something more, or less, than I really am. “Can I, or can I not, free myself of this behavior?” “Perhaps I can.” But the fact is: there are normative elements in states of mind and types of conduct relevant to being an addict, and these normative elements cannot be reframed in the descriptions of neural inhibitory mechanisms operating in independence of a person’s own self-assessment and biographically reflective reason responsiveness. The key for the addict is to find some way – often with professional help or non-professional communal help – to leverage his remaining powers of agency, first and foremost in relation to his DOC, to stop using the Substance. Sometimes the first choice is to be tied like Ulysses to the mast for a time. But it is an important but underestimated fact that every addict who does not use any longer has done exactly that, moderated or stopped using (15, 16, 43). Such former addicts have rediscovered, reclaimed effective agency. And they are abundant.

## The Moral Failure

Persons enter the world valuing certain things and not others and they exit the same way. We are creatures with ends. Some of these have to do with resource needs and acquisitive desires related to these needs – for food, clothing, and shelter; others have to do with social needs, with needs for company and affection. We are gregarious animals. No person, no matter what her conception of flourishing or well-being would choose a life without friends, says Aristotle^13^. When Strawson (18) calls attention to the reactive attitudes, the suite of emotions and sentiments that guide inter-personal commerce and that involve reactions to the good will, ill will, and indifference of others, he is careful to include affection, love, gratitude, and forgiveness, along with anger, resentment, and shame. According to Strawson, these emotional dispositions come with the equipment. He compares the reactive attitudes to induction. We cannot ask whether induction is rational. It is arational, part of our animal nature, not something we can give up. What we can do, however, in both the case of induction and the case of the suite of reactive attitudes is to adjust, moderate, modify, tune up and/or tune down as necessary both natural innate attitudes. We modify our original disposition to apply the straight rule of induction via feedback from its application. For example, when we apply the straight rule to small or unrepresentative samples we get poor predictions and we adjust the rule^14^. Eventually over world historical time, the methods of inductive logic, statistics, and probability theory develop. With respect to the reactive emotional attitudes, different social ecologies develop different norms for apt emotions (19).

A key idea in Strawson is that the reactive attitudes are not only essential to inter-personal relations, they are also essential to how we see, judge, and regulate our own mind and behavior; they are also intra-personal. I can experience, indeed I do experience, the reactive attitudes to my own mental states and actions. Anger at myself for what I did, as well as disappointment, pride, embarrassment, shame, and guilt are familiar components of a human life. Self-esteem is a general feeling that one is decent, worthy, doing well; self-respect involves knowing with some degree of confidence and proper humility that this feeling is warranted.

It is commonplace for modern people to think that ancient and superficial peoples ran on shame whereas we run on guilt. Williams (20) has turned this idea on its head. The idea that, for example, the Greeks were a shame culture not a guilt culture is true but not a weakness or superficial characteristic. Shame is not simply a feeling caused by being seen, naked as it were, by others. It also involves not passing one’s own survey. “Shame looks to what I am” [(20), p. 92]. Guilt, the modern emotion, is the narrow reactive attitude. It is largely internalized anger at certain actions and its roots are in what Williams calls “morality, the peculiar institution.” Morality, the peculiar institution, is the narrow normative domain that encompasses all and only the domains that the God of Abraham is interested in assessing each person on come Judgment Day (its secular version comes in Kant). Ethics, in the broad sense, prized by the Greeks, by Nietzsche, and by Williams, is concerned with living a good human life more generally. It involves aspirations to flourish, which involves living at the intersections of what is good, true, and beautiful, whereas modern moral philosophy focuses primarily on the good, and even there it is narrowly conceived.

Here is how this relates to addiction. Almost all addicts experience failures of basic agent capacities, for example, in the first criteria of DSM 5 there is a failure to do what one reflectively intends. The non-addict will get that the addict might fail if a drink or drug is right in front of her (we relate from chocolate candy type experiences). But the addict will decide, indeed she will resolve not to purchase alcohol or cocaine and then find herself driving to the liquor store or crack house. This is shameful and is experienced as such both on the way to score, although in something of a blur, and afterward. I am ashamed of who I am, not simply for what I did. And it builds. An addict is someone, who like everyone else, has educational, career, and inter-personal aspirations, and he reliably fails to achieve them; or he achieves them to some degree, and then his addiction undermines these accomplishments. Every alcoholic and every addict in rooms of AA and NA and most every memoir of addiction (even if the author is not inculcated into 12-step ways of speaking) will speak of extreme feelings of shame for who one is, who one has become in one’s own eyes, even if one has not yet been fully seen by others and even if objective failures are still in the “not-yet” category (2, 13, 21)^15^.

The main point is that the ongoing epistemic-interpretative failure that involves: (1) the inability to draw normal inferences about the harms one is doing to oneself or others (caused by various classical defense mechanisms); and (2) the inability to get one’s mind, body, and behavior to respond to decisions and resolutions in the normal ways; PLUS (3) the persistent failure to live as one expects oneself to live as a worthy human being undermine the basic goods of self-esteem and self-respect. One is ashamed of who one is. The alcoholic needs to put the “plug in the jug” (the crack addict needs to stop scoring eight balls; the smack addict to put down the needle). But the disease of addiction doesn’t end there and then. And the reason is simple. Addiction is not a synchronic disorder that ends with the end of taking one’s DOC. It is a diachronic molar person-level disorder and as such requires psychological, epistemic, moral, and narrative healing and reconstruction.

## Social Capital

A consistent finding in the literature on human well-being is that the best predictor of well-being – better that income, better than health even – is social capital (22). Almost all the variance between Northern Europeans and North Americans on the one hand, and citizens of sub-Sahara Africa on the other hand, in well-being measures has to do with the fact that almost half of informants in sub-Sahara Africa say that if they fell off a bar stool (here used only metaphorically), there would be no one they could count on to help, not a friend, not their mother, father, brother, or sister.

In his important book, *Bowling Alone*, Putnam (23) plots some of the causes and consequences of breakdowns in community and loss of social capital in America. There are insights for those concerned with addiction in these sorts of studies. First, addictions increase when there is socio-economic displacement, breakdown in community, and the availability of drugs and alcohol. Second, healing individuals typically involves reintegration into community, often a community whose other members have also experienced the bewildering twin normative failures and the self-degradation that results, and who get, at a minimum, that this sort of thing can happen to otherwise decent, worthy people, and who have experience, strength, and hope to share about how to regain control of one’s self, one’s life. Eventually, actually at the same time, there is reintegration into the wider social community, doing school or one’s job as one is supposed to, being there for one’s friends and family in the way a good person is, an end to actual or psychological isolation and concealment that is a common accompaniment of addiction (2, 13, 16, 21, 24, 25). Self-esteem and self-respect return and shame dissipates, possibly pride grows.

## Responsibility “Without the Sting”

Strawson (18) writes about the possibility of taking “the objective attitude” toward certain persons. The objective attitude is one that involves a surmise, possibly a conviction that the normal reactive attitudes are not deserved in certain cases and should be suspended. Children have temper tantrums and anger is not warranted. So we suspend, or try not to act on anger, even if we can’t help to feel it to some degree. We also can and do suspend or try to suspend our normal reactions to the insane, to those who suffer from compulsions, who have no rational control over their actions.

Can and should we take an objective attitude toward the addicts in our midst? Probably. Can or should addicts take an objective attitude toward themselves? Probably. But there are psychological limits to our abilities to overcome natural dispositions. Furthermore, the addict feeling shame and the wider community thinking it is a shame that his life is going so badly is a humane reaction. It need not be taken to warrant blame. It signals that both the addict and we recognize that he could do better and be better. Understanding that he is an addict is a humane way of saying that we get that he is in a terrible fix and that we sympathize (46–48).

The more we learn about the complex socio-psycho-biological nature of addiction, about the ways various cultures encourage heavy drinking, about the effects of SES and drug availability, about genetic propensities, about the effects of weird reinforcement regimens, and of brain glitches, we have reason to adjust full normal subjective engagement to the addict. Williams makes this interesting point: “What arouses guilt in an agent is an act or omission of the sort that typically elicits from other people anger, resentment, or indignation. What the agent may offer in order to turn this away is reparation; he may also fear punishment or may inflict it on himself. What arouses shame, on the other hand, is something that typically elicits from others contempt or derision or avoidance” [(20), pp. 89–90]. This seems right; the life of the addict is a source of both guilt and shame. And thus he receives an odd admixture of reactions from others; in part, there are the normal reactive attitudes that full blown autonomous agents receive, anger and indignation; but there is also something else, a set of reactions that indicate that you have put me – us – off. You are puzzling, weird, to be avoided^16^.

Williams goes on: “His (the person who is ashamed) reaction is a wish to hide or disappear, and this is one thing that links shame as, minimally, embarrassment with shame as social or personal reduction. More positively, shame may be expressed in attempts to reconstruct or improve oneself” [(20), p. 90].

## Conclusion

All this seems about right. And in particular: shame is partly constitutive of addiction. The addict cannot pass his own survey. He is appalled by the twin normative failures from which he suffers, and shame is the appropriate, respectful, humane, first-person response to these failures. Shame begets using and more using begets more shame, and the vicious cycle is produced and maintains itself. Overcoming shame is part of overcoming addiction. Shame is also normally a crucial factor motivating the addict’s attempt to reclaim, reconstruct, and improve himself. It motivates the addict to want to get a grip. That said, there are many reasons for the addict to forgive himself and engage in the difficult project of reconstruction and improvement with the knowledge that his agentic capacities in relation to the Substance are compromised, deficient; and, at the same time and for the same reasons, there are reasons for others to keep the addict in the realm of the very usual, the puzzling, the not-so-nice-to-be-around, but to also engage him with sympathy and compassion, maybe with forgiveness. The more we know about addiction the more this becomes both possible and sensible. At the same time, both the addict and the community that is asked to understand and treat him with compassion need to acknowledge that the addict is a person who suffers twin normative failure. He will need to heal to once again be treated as a full-fledged normal agent. He must regain his full normative agency and regain traction in his quest to live well.

## Four Objections and Replies

### The willing addict objection

The process or condition that you are calling addiction really includes matters characteristic of a certain kind of addict, a so-called unwilling addict. Some addicts, however, are willing, and do not feel shame or guilt over their addictive behavior patterns (26). Willing addicts don’t double back on themselves and wish that their behavior was otherwise or that they should control their impulses to consume. Pickard (25) offers a powerful version of this objection in correspondence. Based on her clinical experience and standard DSM understanding, she writes that among addicts “are some people who are severely personality disordered, really genuinely don’t want people or friends in their lives (this is part of having schizoid PD, diagnostically) and have a ‘relationship’ with drugs instead; some are so narcissistic and grandiose that the claim that they feel shame or look on their lives critically or think they could do wrong would require very deep, very inaccessible levels of the unconscious to make it true.”

The objection is that there are certain individuals with schizoid personality disorder, perhaps there are others in the manic phase of bipolar disorder, who are addicts in the sense that they have lost rational effective control over using, but do not feel shame, and thus do not suffer the twin failures and who thus are not, according to me, addicts. But they really are addicts. So the twin normative criterion is descriptively false.

Before I respond to the objection, I can strengthen it as an objection, by pointing to two recent memoirs where the protagonists might be addicts in the sense of satisfying condition no. 1 of the twin normative failure model – he can’t stop if or insofar as he tries – but he doesn’t feel shame. *Narcopolis* by Thayil (27) and *The Wet and the Dry* by Osborne (28) brilliantly present two different types of character who don’t seem to satisfy the shame condition. In Thayil’s semi-fictional memoir, the 1970s opium dens of Mumbai, then known as Bombay, are a romantic haven for souls who have almost no other options, plus opium is really cheap. He and they are addicted and they don’t give a shit. Even if Thayil is not proud to be an addict, he is not ashamed either. Osborne’s story meanwhile is a hilarious romp through the Middle East by a man who is a “drinker” and who is hoping that laws and social mores of Muslim countries that disapprove of drinking will help him at least temporarily to moderate. They don’t and he doesn’t.

So now the strengthened objection is this: there are at least four types of addicts that do not satisfy the shame condition: (1) people with personality disorders such as schizoid PD; (2) people in full blown mania; (3) people that have easy access to the Substance, to their DOC, and no other choice-worthy options are available [see Ref. (29, 30)]; (4) “Drinkers,” like Osborne – also think of Richard Burton, Richard Harris, Peter O’Toole, and Christopher Hitchens or a heroin user with resources to get reliably pure doses, a Keith Richards type. This latter may be an approved of life style among a certain mostly white elite in the UK, but probably not in the US.

#### Reply

No doubt cases of addiction are heterogeneous in many respects, and dimensional in depth, severity, and so on. Are there willing addicts?^17^ Surely, there are people who minimize and rationalize, and people who think they could stop if they decided to do so. Some of these probably do fall into the class of willing users, even willing abusers. They choose to use, but believe they could stop if they had sufficient reason to do so. They like using excessively, asocially, possibly even antisocially. Some of these people might be wrong that they could stop if they tried (in the normal reason-responsive way), in which case they would be wrong that they are not addicts because they do not satisfy the first normative condition, the effective agency condition. They do; they just don’t know that they do. The *Big Book of AA* says if you think you are not an alcoholic, you may be right. There is a test: try some controlled drinking and if you can do so reliably and without always feeling overwhelming desire to use, then you were right – you were just a heavy drinker not an alcoholic. On my view, if a person could stop if they decide to do so, they do not suffer the first normative failure of effective agency (nor would they be self-deceived, etc.). And such an individual would not be an addict according to the view on offer.

But what about the memoir cases and Pickard’s psychiatric case(s)? One thing to say about the two memoir cases is that in both cases some shame is experienced; Osborne, at least, is often embarrassed about the blackouts and some of the predicaments his drinking gets him in. The shame condition in the twin normative failure model does not specify how much shame needs to be experienced. This could also be said of people with bipolar disorder when they are not in the grandiose bullet-proof phase, during which down-times they do backtrack, second-guess, and so on.

But this doesn’t solve the problems with the Pickard case of schizoid personality disorder where no shame is experienced diachronically; where possibly there is pride instead. A response specifically to these cases might distinguish addicts who satisfy the first condition (call them addicts type-1) and those who satisfy both (addicts type-2). Another is to claim that even the people who don’t feel shame ought to, which concedes that the criterion is normative not descriptive. The option I am inclined to take is to restrict the twin normative failure model to people who do not have severe personality disorders (e.g., schizoid PD) and people who are not in the grandiose phases of bipolar disorder. This would still leave the model open to this objection: there are social environments that are so degraded that there is no shame in addiction descriptively – perhaps the addicts are literally and rightly hopeless – and in which shame, guilt, blame are normatively unwarranted. Shamelessly addicted is simply the way some people live. I accept this. In such environments the concept of addiction in my sense has at best only a weak grip, only the first condition of addiction would be met, and even that only in a weak sense: if the resigned or hopeless addicts in such worlds wanted to stop (they don’t), they couldn’t^18^. The twin normative failure model is a useful model in environments where there are multiple choice-worthy options, not otherwise.

### The essence and the periphery objection

The twin normative failure model of addiction is swollen and inflated. There is too much that you are including as proper parts of addiction or of an addictive behavior pattern. The essence of addiction is at the brain level. The presence or absence of shame and negative self-evaluative attitudes, of various moral attitudes and emotions, the failures of reason responsiveness are sequelae of addiction, not part of addiction.

#### Reply

First, if an essence involves characterizing the set of properties that are invariant or at least highly reliable accompaniments of a kind, then the two normative failure model has at least as much credibility as any other model. I claim that you will find evidence of both normative failures in most every addict’s first personal testimony and in the third personal testimony of professionals who work with addicts. The shame of addiction is shame that is directed to the content that [I cannot control my behavior in relation to the Substance] and that [because of my using the Substance I fail to live up to my ends, values, goals, and standards]. Those who favor only brain or genetic bases for the disease have yet to agree about what that single basis is, if there is one, or whether it is polyneural or polygenic, if there are several, and how exactly (and when) confirmation/disconfirmation might come for the various contender hypotheses. I claim that there is confirmation for the twin normative failure model right now. Second, although a less inclusive or thinner concept of addiction (a least common denominator conception, as it were) may work as an operational stipulation in the case of models of certain non-human animal behavior, it is without merit in the richer conceptual and normative world of human beings, at the person level. Third, and relatedly, the objection favors an unrealistic simplifying assumption requiring that we define the dysfunction of addiction synchronically rather than diachronically, and over some aspect that is hypothesized, but not yet shown to obtain over all creatures that can suffer addiction. But claiming that the hypothesized shared basis is the essence, and that all other features, especially ones that reliably appear in *Homo sapiens* are not, is to change the question. Fourth, the method of gaining insight into essences is unstable. We have already seen how some geneticists think they can reduce the neuroscientific base of addiction to a genetic one in DNA, and even RNA, that serve the salt or water instincts (31) ^19^. For familiar reasons, this move opens the geneticists’ account to a further reduction into the language of bosons and fermions or whatever is the language of fundamental physics. With each reduction we move further and further from the phenomenon we started and are providing a less ecologically valid account of that phenomenon. If we are speaking of addiction among humans and addiction constitutes any sort of well-behaved or unified kind, every bit of evidence indicates that it is a psychological or behavioral kind that is also a double-normed social kind. This is perfectly compatible with this kind also having certain common features at the level of the brain and genes since kinds defined at the higher level have all the properties that the lower levels have but the reserve is not true, and this matters.

### The intervention objection

The key to curing addiction is to arrest it, to stop the addict from using. Your view says that addicts fail to be able to exert normal self-control capacities and are ashamed of both this fact and the fact that they are failing to live a good human life. But you also acknowledge that knowing or experiencing this and also desiring to stop is normally not enough to stop. For this we are working toward pharmacological interventions that help addicts stop using, which is a necessary condition for any and all further healing. There are drugs that make the alcoholic sick if she uses, and others that mitigate the effects of cocaine and opiates. Eventually, work in genetics will yield simple interventions that adjust genes for those predisposed, and so on. If you think these interventions are already working, or might work, to arrest addiction, then you acknowledge that brain or genes cause of addiction.

#### Reply

The first part of the objection is not an objection to the view. I have said exactly nothing about opposing any and all therapeutic techniques that are helpful to the addict. If various kinds of psychopharmacological interventions can help without comparable costs, then, good, use them. But do not make the mistake of thinking that in locating an intervention site that one has identified the cause. Also beware the related mistake (sometimes made by psychoanalysis) that the root cause must be treated to arrest an ailment. First, many things are fixed without fixing what caused the breakdown. The weather caused the bicycle chain to rust. I clean the chain and oil it. I fix the bike but have done nothing to the weather. On the other side, we need to beware mono-causal thinking. The pragmatics of causal talk makes it sensible to say such things as the rock broke the window. But really the rock only broke the window because it, the window, had a certain density and brittleness (if it had been shatterproof, no breakage). And the window broke only because the boy threw the rock, and he threw it only because he was angry, and so on. No rock, no broken window; no angry boy, no broken window. The best analysis of causation in the philosophy of science says that the total cause of Θ is the set of events and processes (α, β, γ, δ … ω) such that if (counterfactually) anyone of them were different Θ would not have occurred as it is (32). And so it is with addiction. Take away the family that thinks adult drunkenness or drug use is amusing and some “addicts” never become one, take away the hopelessness of some urban environments and the rate of addiction will go down, and so on for genetic predispositions, etc. The first point is that many interventions do treat or require treating causes, and in so far as it is wise to treat a cause (or constituent or effect) of some disorder, and such intervention is effective, this should be applauded. But it is not at the same time any evidence at all that the cause has been found. It is rare for any phenomena that there is any such thing as the cause. Genes are causal factors in addiction, brains are causal factors, and families are causal factors. But invariably using a fair amount of some substance is a causal contributor to addiction, and this social practice – drinking, snorting, and mainlining – is not in the genes or the head.

### Objection: Re-moralizing addiction

Fourth and finally, it will be objected that the appeal to the shame of addiction reintroduces the idea that addiction is a moral failing.

#### Reply

This is a simplistic and mistaken objection. Addiction is a normative disorder, a twin normative disorder that involves shame at one’s own survey, first because one is not an effective agent in relation to the Substance and cannot reliably do what one judges best, and second, because one is messing up one’s life because of one’s relation to the Substance. These are normal responses by the addict to his own realistic assessment of his plight. It is an interesting and important question whether an addict can take or adopt an “objective attitude” toward himself. It seems often to occur to some imperfect extent, and insofar as it does happen it may well prepare the person for self-forgiveness, and for reclamation of self-esteem and self-respect. There can be shame without blame. We acknowledge this when we say of the addict or the way he lives that “it” is “a shame.” Or to put the matter another way: guilt is anger turned inward and normally involves blame. Shame involves disappointment at self, but need not involve anger at self. Anger can immobilize; but shame can and often does motivate a change in direction, a search for a way to overcome his extraordinarily destructive relation to the Substance. As for others, knowledge is power, and the more that is know about the nature, causes, and multifarious trajectories of addiction, the more reasons others have to treat addicts as special cases, not as suffering an ordinary physical disease, but also not as fully effective agents, as worthy of sympathy and compassion because they suffer the shame of addiction. My recommendation is to accept that addiction just is a normative disorder, while at the same time not moralizing it.

## Conflict of Interest Statement

The author declares that the research was conducted in the absence of any commercial or financial relationships that could be construed as a potential conflict of interest.

## Footnotes

^1^I mean the view to apply in the first instance to alcohol, cocaine, and opiate addiction.

^2^According to Dennett’s pragmatic taxonomy, the “intentional stance” is person-level psychology; the “design stance” is computational cognitive psychology, and the “physical stance” is neuroscience. One could, for pragmatic reasons and perfectly in the spirit of his taxonomy go higher than the intentional stance – to sociology and then anthropology – and lower that his physical stance, to biochemistry and eventually to basic physics. One my view, but possibly not on Dennett’s (he is well-known for his instrumentalist or eliminativist tendencies), the higher-level entities truly have properties that the lower levels don’t have. There really are such things as beliefs and desires. People have beliefs and desires; people contain brains and brains contain neurons; but neurons don’t have brains and brains aren’t people, and probably brains do not have beliefs and desire, although people with brains do, and so on.

^3^I don’t mean to overstate the degree to which people do or ought to examine and evaluate their lives from an articulate reflective pose (see Flanagan (33), for a critique of Charles Taylor for this intellectualist mistake). The point is that people have ideals, ends, goals, and purposes – many of which are socially scripted – and we are consciously or semi-consciously aware of how we are doing in relation to these ideals, ends, goals, and purposes. There are rational evaluations and adjustments of both ends and means along the way, as well as all the familiar kinds of rationalization and defensive denial that one really wanted to do such and so, or be such and so in the first place.

^4^“Self-represented identity” is the story from the first-person point of view; “actual full identity” is the true story of who one is, what one is like, the story that would be told by an ideal observer with the right theory of the human mind and action and knowledge of all the facts.

^5^I need to emphasize the idea that evaluation occurs “in broadly moral terms.” Later, following Williams (34), I distinguish between “morality, the peculiar institution,” which is roughly the kind picked out as “moral” inside the Abrahamic traditions and “the ethical,” which is much broader and includes the aspirations to live well by achieving maximal intersection of the goods that comprise what is true, beautiful, and good. I intend what I am calling “moral” and what constitutes “the moral failing” of the addict to align with “the ethical” and not with “morality, the peculiar institution.”

^6^The *Big Book of AA* describes the relevant first-person phenomenology as “incomprehensible demoralization (p. 30).”

^7^I am collaborating on a project with geneticists (31) who find that in mice, cocaine and opioid use activates genes associated with the salt and water instincts. The instincts to maximize salt and water intake when these are present and the organism needs them, are clearly adaptations, perhaps in exactly the form that leads some, but by no means all mice (29, 30) to become addicted mice. The addiction that piggybacks on these instincts is not an adaptation; it was not selected for in the original evolutionary environment or in recent ones to serve a fitness-enhancing effect. Liedtke thinks that this gene-level activity may be what at the genetic level subserves what some neuroscientists call the “midbrain mutiny” that involves the “hijacking” of normal reasons-responsiveness and control capacities by unusual schedules of reinforcement (35), or by an unwelcomed disassociation of the normally coordinated brain based “liking-wanting” systems (36), or by exhaustion of the brain’s mental muscle, aka, “willpower” (37), or by stress hypersensitivity (38). This gene-level explanation of higher order brain level processes that subserve addiction, which itself rests on an evolutionary explanation of selection of genes that code for salt and water instinct, and (45) one of these higher order brain explanations can both be true as far as what happens at the gene level and the brain level without even the combined resources of both levels remotely describing or explaining addiction at the person level.

^8^“The reactive attitudes” according to Strawson (18) are the set of familiar sentiments, emotions, or attitudes such as anger, guilt, shame, forgiveness, resentment, happiness, and gratitude that regulate human interaction.

^9^Insell (39) the head of NIMH is impatient about filling in the details, and wants to push on to the neuro-specifications of all bona fide mental disorders quickly. And he is willing to put the money he controls as head of NIMH where his mouth is Meanwhile, Frances (40) former head of the DSM IV taskforce laments the rush to neuroscientific reduction because neuroscience is immature, and because, like me, he is skeptical that all mental disorders are brain disorders, and third because he worries about the dominance of money in seeking neuro-pharmacological “cures” for problems that either are not in the brain at all or, even if they have a brain dysfunction component, are mostly psychosocial problems.

^10^Ahmed (29, 30) has done very important work inside the animal modeling tradition emphasizing that most rats offered cocaine do not get addicted and, more importantly for treating addiction, that compulsive cocaine use is lower in environments where there are multiple pleasure-producing and choice-worthy options besides the cocaine. My concern with animal models is not based on denial, not even skepticism, that there are genetic and/or neurobiological components of addiction, or on doubt that the brain might be the best place for interventionist strategies to short circuit the desire to use, the compulsion to use, the effects of use, and so on. It might be. The point is that even if the brain is an effective, even the most effective, site of intervention, this is not evidence that addiction is a brain disorder or that it is sensible or good to model it as such. Removing the battery of a car is an excellent way to immobilize it, but a running car is not a battery phenomenon and is not well modeled by the battery, what the battery is, does, and the ways it can malfunction. See below on Woodward’s (32) model of causation for why this is so.

^11^Contrast DSM 5 (12) with Graham’s (9) – what I call “the ignoble eightfold path of addiction,” especially, (iii), (iv), and (vii) which emphasize the self-directed reactive attitudes in addiction. DSM 5’s 1–11 speak about ways the addict fails to exert various kinds of control despite his best efforts and it speaks about feelings toward alcohol (craving, tolerance). I am emphasizing the addict’s feelings toward his SELF, possibly as a result of these failures and the harm he does to himself and others. And these reactive attitudes I claim, are part of what addiction is, and what needs treatment. TWO CAVEATS: first, in (vi–viii) Graham places I think too much stock in the belief that relapse is more or less inevitable. Second, I read (vi) as descriptive not prescriptive. It is true that relapse powerfully engages recognition of the twin normative failures including shame and its suite. Increased knowledge about addiction, of course, teaches that despite the shame, the addict may not in any robust sense be to blame or deserve blame.

(i) A person commences a behavior that is potentially harmful or deleterious. They consume a deleterious drug or other chemical substance or gamble.
(ii) The behavior eventually becomes an object of focal attention and periodically repeated or habitual activity. The focus is such that at times or in some cases it may be “the only tune or story in the addict’s head, and nothing else drives it out”.
(iii) The behavior produces consequences that are not just harmful or that seriously risk harm to self (and/or to others) but are perceived as harmful or destructive by the agent.
(iv) The perception or self-conscious experience of harmful consequences leads the person on certain lucid, critical or self-reflective occasions to negatively self-evaluate the behavior and attempt to refrain or quit. This does not mean the addict knows how to quit or how difficult it may be to quit or whether they can quit for any consistent period of time. But an attempt to refrain is made.
(v) The addict refrains, quits or inhibits the behavior during certain periods (perhaps without assistance, perhaps only with assistance – individual cases and occasions vary).
(vi) Quitting or cessation ultimately (timing and intervals vary) proves unsuccessful, however. The addict relapses. They “fall back” into the detrimental behavior after a period of temporary stoppage. The behavior returns together with its negative consequences or risks.
(vii) Relapse is interpreted by the agent as a form of personal disappointment or failure, not just as something destructive or risky, but as a source of shame, regret, self-blame, and embarrassment or as grounds for diminished self-confidence or self-esteem.
(viii) The steps or phases of harmful behavior, temporary abstention, and relapse cycle repeatedly. The recycling, in some cases, may cease permanently, perhaps without harmful long-term residue. And the person just plain quits. (Thousands of addicts just plain quit for good at some point. They “age out” of their addiction.) Or the addictive pattern may lead to an addict.s enduring exposure to harm or personal demise.

^12^One can, of course, make a rodent a psychological mess, extremely anxious, fearful, and so on, by mixing reinforcement schedules that both encourage and inhibit addiction.

^13^Psychopaths or people with schizoid personality disorder might be exceptions to Aristotle’s surmise.

^14^The straight rule is crude and says this: if I observe that A’s are B’s to m/n, then I do/should infer that unobserved A’s are B’s to m/n.

^15^The 12-step community speaks of addiction as a medical, psychological, and spiritual disease. Amazingly, the medical aspect was once thought to be or to involve an allergy to alcohol. The psychological and spiritual aspects refer to a host of problems in the self-esteem, self-respect, shame, and self-degradation arena.

^16^Pickard (25) argues that for individuals with both addictions and personality disorders, we can and should decouple (1) Holding a person responsible for what she did; (2) Holding her responsible for her future actions; (3) Blaming a person for failures on 1 or 2.

^17^Kennett (41) helpfully distinguishes four possible kinds of addicts: willing; unwilling; wanton; and resigned. She is skeptical about willing addicts. Wanton addicts, if there are any, are not reflective in ways that would allow noticing the twin normative failures or perhaps if they did, caring about them. Mice addicts are wanton addicts. I doubt that there are any person addicts who fit the bill since even wantons will need to schedule desires and trying to do so will result in recognition of their inability to successfully schedule substance-related desires (unless supply is unlimited), and thus in recognition and bewilderment over their control capacities. This much will lead, even the amoralist, to frustration and anger at his failure to live as he wishes, to coordinate demand/need to supply, and to gain the satisfactions he seeks. There probably are resigned or hopeless addicts, but even they will normally experience themselves, and will be seen by others as suffering the twin normative failures of loss of effective agency and life standard failure, although the space of live options for such resigned individuals will have pretty much closed off. Resignation is normally a sad emotion constituted in part by the sense that things could have gone better. When an environment is really objectively hopeless, offering no decent options for a good human life, then there just may be no norms against which the addicts life is better or worse than any other. I accept that in such environments, the twin normative failure model starts to lose its grip; perhaps, it is irrelevant.

^18^These sad cases are “resigned addicts” (41). It needn’t be that a whole social world has no choice-worthy options; it can be that certain social groups are trapped by racism or sexism or terrible poverty to have no or very limited choice-worthy options in a world where there are abundant options for others.

^19^To be fair, since full blown reduction never occurs, the geneticist should not claim reduction, but something like the further specification of mechanisms at the lower level (genes) that explain partly why the brain is doing what it is doing. Similar humility on the part of those who work on the neuroscience of addiction would claim to provide insight into some of the mechanisms involved in addiction.
